# Advances in Cardiovascular Therapies and Their Unintended Consequences

**DOI:** 10.1016/j.jscai.2024.102433

**Published:** 2024-11-15

**Authors:** Bina Ahmed

**Affiliations:** Department of Cardiology, Cottage Health, Santa Barbara, California

**Keywords:** cath lab, climate change, recycle, sustainability, waste

Unintended consequences of an action can arise in complex systems. Sometimes, they are the result of focusing on short-term gains without considering the long-term effects. Other times, they are accepted as a necessary outcome of accomplishing the primary goal. Furthermore, attempts at mitigating the effects of unintended consequences are often thwarted by the perceived impossibility of implementing change to the complex system.

Climate change is an unintended consequence of anthropogenic influences. Similar to other major economic sectors, all aspects of health care make a sizable contribution to carbon emissions and with it climate change. Every step in the complicated process of treating each patient leaves a globally measurable footprint and without direct intention contributes to health care’s carbon footprint. Given its size, it is not surprising that the global health care system is the fifth largest contributor to greenhouse emissions worldwide.^1^ Until recently, measuring, understanding, and mitigating health care’s carbon footprint was not an achievable priority, but as the scale of the problem comes into focus, so has the need for action.

In the current issue, Amin et al^2^ report their findings of an internal audit of the amount of hazardous and nonhazardous medical waste produced from the cardiac catheterization laboratory (CCL) and cardiac operating theaters (COT) in a tertiary cardiac care center in Bahrain. First, the authors are to be commended on doing this important work. There are specific challenges and deterrents that come with studies quantifying utilization and output related to contaminated after-use products. Their study is also unique in that it evaluated the quantity of contaminated and recyclable waste from procedures performed in both the CCL and the COT. They audited the amount of hazardous and nonhazardous medical waste produced from their CCLs and COTs over 2 months. Based on their findings, they estimated that their practice generated 11,000 kg (24,200 lb) of recyclable waste and 30,000 kg (66,000 lb) of contaminated waste per year. If this is extrapolated to all the CCLs and COTs globally, the anticipated annual waste production from invasive cardiac procedures is 150 million kg (150,000 metric tons or an unimaginable 330 million lb) or the weight equivalent of 15 Eiffel towers or approximately 1000 blue whales.

Other notable findings highlight different mechanisms of carbon output from managing different types of waste. While nonhazardous waste will clog landfills, hazardous medical waste needs to be processed using more intensive methods of either incineration or autoclaving before reaching the landfill. Coronary artery bypass grafting plus valve surgery had the highest output, with each case generating 9.4 kg of recyclable waste and 31 kg of contaminated waste (total 89 lb). Transcatheter mitral and tricuspid valve replacements were also high producers, with each case amounting to 24 kg or nearly 52 lb of waste. Even simple procedures like coronary angiography generated about 5.6 kg or 12 lb of waste per case. The benefit of assigning a numerical value such as weight to each procedure, as done diligently by Amin and colleagues in the current study, also quantifies the potential impact of implementing decarbonizing practices. For example, aiming for a 50% reduction would result in 115 million pounds of CCL and COT waste being saved globally.

The authors go on to discuss reasons and ways for us as part of the global health care system to lead the charge and forge the path for change. There is much that can and needs to be done. Developing a sustainability team or task force for the CCLs and COTs is essential. Identifying specific and implementable ways on both the microscopic and macroscopic levels will take a committed team effort. For those in the CCL or COT, opportunities to reduce, reuse, and recycle whenever possible are abundant (and woefully underused). In addition, there has to be buy-in at the level of hospital leadership. Leadership is often the most removed from the process of delivering actual patient care and may need to be convinced about the importance of not just decarbonizing the procedural rooms but also for ecological sustainability to be a core tenet for the entire health care system. If procedural areas are to capture all potential recyclables, the hospital system must have pathways to reliable recycling.

There is also a need to intensify efforts to raise awareness. Some might argue that given the burgeoning threat of climate change on human health, algorithms and plans to decarbonize health care are as important as protocols to treat common ailments. Bringing health care sustainability sciences into the medical school and resident curriculum will allow for more innovative, imaginative, and critical thinking championed by the generations that follow.

As we discuss how best to address/mitigate/improve the unintended carbon footprint of “saving lives,” there is a natural sense of feeling lost and overwhelmed by the complexity and/or impact of the solution. Whose responsibility is it? What can the impact of saving a few pounds of waste per case be when there are more egregious acts of carbon spewing that remain unregulated? While those are understandable arguments, they fail us, our patients, and the principle of “primum non nocere.” Advances in cardiovascular therapies have allowed our patients to have access to life-changing and often life-saving therapies. But in its current form, it comes at a cost. To do best for our patients (and humanity), we have to consider all the consequences and all the potential harm. A life saved today should not come at the cost of lives lost to the devastating effects of climate change and unfettered carbon emissions for decades to come.

“I am only one but still I am one. I cannot do everything but still I can do something and because I cannot do everything, I will not refuse to do something that I can do.”

–Edward Everett Hale
